# Listeriosis in Pregnant Women and Neonates in China: A Systematic Review

**DOI:** 10.3390/jcm15155915

**Published:** 2026-07-29

**Authors:** Baorong Gao, Yali Miao, Rui Miao, Hui Ye

**Affiliations:** 1 Department of Obstetrics and Gynaecology, West China Second University Hospital, Chengdu 610041, China; 2 Key Laboratory of Birth Defects and Related Diseases of Women and Children (Sichuan University), Ministry of Education, Chengdu 610041, China; 3 Department of Medical Oncology, Key Laboratory of Cancer Prevention and Intervention, Ministry of Education, The Second Affiliated Hospital, School of Medicine, Zhejiang University, Hangzhou 310009, China

**Keywords:** listeriosis, pregnancy, China, epidemiology, systematic review

## Abstract

**Background:** Pregnancy-related listeriosis typically causes mild, self-limited maternal symptoms but can lead to severe fetal and neonatal outcomes, posing a significant public health concern given its rising incidence. However, data on the epidemiology of this infection and its impact on maternal and neonatal outcomes in China remain scarce and fragmented. **Methods:** A systematic search was conducted in the following three Chinese-language databases (CNKI, Wanfang, and CBM) and two English-language databases (PubMed and Embase) for studies performed in China from 1 January 2018 to 14 March 2026. Relevant studies published before 2018 were identified from two previous systematic reviews. Information on the epidemiology, clinical manifestations, and outcomes of pregnancy-related listeriosis was extracted. For inclusion in pooled incidence estimates for pregnant women, studies were required to have the number of pregnant women or pregnancies as the denominator; for pooled neonatal case fatality risk estimates, studies were required to report the total number of neonatal listeriosis cases. The methodological quality of each included study was appraised using the Joanna Briggs Institute critical appraisal tool. Heterogeneity among the studies was quantified by the *I*^2^ statistic. All statistical analyses were performed using STATA version 12 and Microsoft Excel 365 (PROSPERO: CRD420261326136). **Results:** Of 389 abstracts initially identified, 160 full-text articles were retrieved for detailed evaluation, and 73 studies (including two previous systematic reviews) ultimately met the inclusion criteria. These studies, which were conducted across 19 provinces, provided sufficient data for analysis, encompassing 956 pregnant women and 749 neonates. Pregnancy-related listeriosis showed regional variation, with Beijing (*n* = 215), Zhejiang (*n* = 143), and Shaanxi (*n* = 142) contributing the most cases. The pooled incidence of obstetric listeriosis was 11.57 per 100,000 pregnancies (95% CI, 7.63–15.50; *I*^2^ = 73%) based on nine studies, and the pooled incidence of neonatal listeriosis was 7.55 per 100,000 live births (95% CI, 4.44–10.67; *I*^2^ = 60%) based on six studies. Fever (53.7%, 513/956) was the most common symptom among pregnant women, whereas respiratory distress (34.8%, 261/749) predominated in neonates. For empirical therapy, only 6.0% (57/956) of pregnant women received a penicillin-based regimen, whereas 32.5% (311/956) received cephalosporins. Two maternal deaths were recorded, whereas major adverse outcomes (miscarriage, stillbirth, prematurity, or low birth weight) occurred in 58.3% of pregnancies. Among neonates, 27.5% (206/749) received empirical penicillin therapy and 15.6% (117/749) received cephalosporins. The neonatal case fatality rate was 11% (95% CI, 0.06–0.17; *I*^2^ = 60%). **Conclusions:** Pregnancy-related listeriosis in China carries a substantial burden, with high rates of adverse fetal and neonatal outcomes. Suboptimal surveillance and inappropriate empirical antibiotic therapy likely contribute to these poor outcomes, highlighting the urgent need for enhanced surveillance, prompt recognition, and appropriate antibiotic use when *Listeria* infection is suspected.

## 1. Introduction

Listeriosis is a rare but severe invasive infection caused by *Listeria monocytogenes* (*L. monocytogenes*), a small, facultatively anaerobic, Gram-positive, motile bacillus [1]. The organism is ubiquitous in the environment and exhibits remarkable tolerance to low temperatures, salinity, and acidic conditions, which facilitates its survival in a variety of food products [1,2]. Most non-neonatal cases are foodborne, particularly associated with the consumption of ready-to-eat or refrigerated products [2]. *Listeria* infection poses a particular risk during pregnancy because the pathogen can cross the placental barrier, thereby infecting both the mother and the fetus [3]. Although maternal outcomes are generally favorable and often manifest as mild febrile illness or flu-like symptoms, vertical transmission may lead to serious fetal and neonatal complications, including miscarriage, preterm labor, stillbirth, fetal loss, neonatal sepsis, meningitis, and congenital pneumonia [4,5,6,7,8,9]. The spectrum of perinatal listeriosis is highly variable, ranging from subclinical maternal infection to life-threatening neonatal disease. Its clinical features are neither sensitive nor specific and may include a combination of infection-related lesions, such as placental abscesses, nodular lesions, and vascular lesions (either ischemic and/or hemorrhagic), as well as brain abscesses [6,7]. The detection of parenchymal lesions is associated with a poor prognosis [7]. This broad and non-specific presentation complicates timely diagnosis and clinical management.

Listeriosis is a globally distributed infection that has become an increasing public health concern, driven by rising incidence rates in several high-income countries over recent decades [5,8]. This upward trend likely reflects demographic shifts toward an aging population, greater consumption of ready-to-eat foods, and enhanced surveillance and diagnostic capabilities [8]. Pregnant women are at a disproportionately higher risk of infection compared with other high-risk populations, such as the elderly and immunocompromised individuals [8], and the infection shows a distinct predilection for the second and third trimesters [4,5]. The underlying mechanisms for this trimester-specific susceptibility remain incompletely understood but are thought to involve gestational changes in cell-mediated immunity and hormonal influences on pathogen–host interactions [3]. However, reliable epidemiological data are primarily available from countries with mandatory reporting systems, such as the United States, European nations, and Israel [4,5,7,8,10]. In contrast, in China, a sentinel hospital network for foodborne disease surveillance was established in 2010 [9]; however, its coverage remains limited to a selected number of hospitals, and case reporting is largely restricted to outbreak-associated episodes, leaving a substantial gap in population-based estimates of the true disease burden.

Most *Listeria* infections during pregnancy are sporadic and can cause fetal harm even in the absence of overt maternal symptoms [8,10]; consequently, awareness of the burden of invasive listeriosis among pregnant women and neonates remains notably limited. Existing estimates are largely derived from small-scale or single-center databases [11,12,13,14,15,16,17,18,19,20,21,22,23,24,25,26,27,28,29,30,31,32,33,34,35,36,37,38,39,40,41,42,43,44,45,46,47,48,49,50,51,52,53,54,55,56,57,58,59,60,61,62,63,64,65,66,67,68,69,70,71,72,73,74,75,76,77,78,79,80,81], which restricts their generalizability to the broader Chinese population and introduces potential selection biases. Moreover, underdiagnosis of mild or asymptomatic infections further compounds the uncertainty surrounding the actual incidence rates. Given that sepsis is a leading cause of neonatal mortality worldwide [82], understanding the specific contribution of listeriosis to neonatal sepsis is essential for prioritizing public health interventions. Two previous systematic reviews on this topic included studies from China only up to 2010 [9] and 2017 [83], respectively, and neither provided a comprehensive assessment of geographic distribution, clinical management patterns, or maternal and neonatal outcomes across different regions of China. This highlights an urgent need for an updated and comprehensive synthesis of the available evidence. To address these critical gaps, we conducted a systematic review with the aim of determining the incidence of listeriosis among pregnant women and neonates, delineating its geographic distribution across Chinese provinces, and summarizing current approaches to clinical management and associated outcomes so as to further identify priority areas for future research and surveillance.

## 2. Methods

### 2.1. Search Strategy

We conducted a PRISMA-compliant systematic review [84] of pregnancy-related listeriosis (Supplementary Materials S7). We systematically searched PubMed, Embase (via Ovid), China National Knowledge Infrastructure (CNKI), Wanfang Database, and SinoMed (CBM) for studies published between 1 January 2018 and 14 March 2026. Search terms were developed iteratively and included multiple variants and Boolean combinations of terms covering three conceptual domains: (1) “listeria” or “listeriosis”; (2) “pregnancy,” “pregnant,” “maternal,” “perinatal,” or “obstetric”; and (3) “infant,” “neonate,” “newborn,” or “fetal.” The search was restricted to studies conducted in China, with no language restrictions applied. The full search strategy for each database is provided in Supplementary Materials S1. Relevant studies published before 2018 were identified from two prior systematic reviews, which included studies up to 2010 [9] and 2017 [83], respectively. For studies published in Chinese, we translated abstracts or full texts into English using an online translation service. The study protocol was prospectively registered with PROSPERO (CRD420261326136). As this study involved no primary data collection from human participants, ethical approval and informed consent were not required.

### 2.2. Inclusion and Exclusion Criteria

Publications were considered eligible for inclusion if they reported at least one of the following types of data on listeriosis among pregnant women or neonates in China: (1) epidemiological data (e.g., incidence, geographic distribution, or seasonal patterns); (2) clinical data (e.g., maternal or neonatal symptoms, signs, or laboratory findings); or (3) details on antibiotic treatment regimens and clinical outcomes. No restrictions were imposed on study design. Exclusion criteria were applied as follows: (1) non-human studies (e.g., animal or in vitro experiments); (2) studies without original data (e.g., narrative reviews, editorials, or commentaries); (3) experimental or interventional studies not reporting clinical outcomes of interest; and (4) case reports describing fewer than five patients, which were excluded to minimize publication bias and to ensure sufficient clinical data for meaningful synthesis-a pragmatic balance between including valuable rare-disease data and ensuring reliable pooled estimates (Supplementary Materials S2).

### 2.3. Definitions

Invasive listeriosis was defined as the laboratory isolation of *L. monocytogenes* from a normally sterile site (e.g., blood, cerebrospinal fluid, or amniotic fluid) in a pregnant woman or neonate. Consistent with the World Health Organization (WHO) definition, a neonate was defined as an infant aged 0–28 days. Early-onset disease was defined as onset at 0–7 days of life, whereas late-onset disease was defined as onset at 8–28 days of life. For data extraction regarding complications in neonatal listeriosis, pneumonia was defined as cases with radiographic findings described on chest X-ray or CT. Neurological impairment (e.g., hydrocephalus, brain abscess, intracranial hemorrhage) was defined as cases with corresponding findings on cranial CT or MRI. Incidence rates were calculated as the number of obstetric listeriosis cases per 100,000 pregnancies for maternal outcomes and as the number of neonatal listeriosis cases per 100,000 live births for neonatal outcomes. To be included in the pooled incidence estimates for pregnant women, studies were required to report the total number of pregnant women or pregnancies as the denominator. Similarly, for inclusion in pooled case fatality risk estimates for neonates, studies were required to report the total number of neonatal listeriosis cases as the denominator. The 95% confidence intervals for the incidence and case-fatality rates were derived separately per study via the Clopper-Pearson exact binomial method, and no transformation was performed. When multiple publications originating from the same study population or institution were identified, we retained the study with the larger sample size or the most comprehensive data to avoid duplicate counting. In cases of multiple-gestation pregnancies, each affected neonate was counted as a separate case.

### 2.4. Data Abstraction and Statistical Analysis

Two researchers (*R. Miao and H. Ye*) independently screened all retrieved records at both the title/abstract and full-text stages using a standardized screening form. Discrepancies were resolved through discussion or, when consensus could not be reached, by consultation with a third investigator (*B. Gao*). From each eligible study, we systematically extracted the following information: first author, publication date, region, study design, data collection period, numbers of maternal and neonatal cases, incidence, and seasonality. The quality of each included study was independently assessed by two reviewers (*Y. Miao and H. Ye*) using the Joanna Briggs Institute (JBI) critical appraisal tool (Supplementary Materials S3) [85]. Clinical characteristics and outcomes—including maternal fever, gastrointestinal symptoms, antibiotic regimens, pregnancy outcomes, neonatal sepsis, meningitis, and mortality—were recorded separately for mothers and neonates (detailed in Supplementary Materials S4 and S5) for descriptive synthesis. All extracted data were entered into a standardized Microsoft Excel template, and data accuracy was verified by cross-checking.

Pooled estimates of the incidence of obstetric and neonatal listeriosis, as well as pooled estimates of the neonatal case fatality rate, were calculated using random effects models. Statistical heterogeneity across studies was assessed using the *I*^2^ statistic, with values of 25%, 50%, and 75% considered to represent low, moderate, and high heterogeneity, respectively. All statistical analyses were performed using STATA version 12.0 and Microsoft Excel 365.

## 3. Results

### 3.1. Literature Search

**Figure 1:**
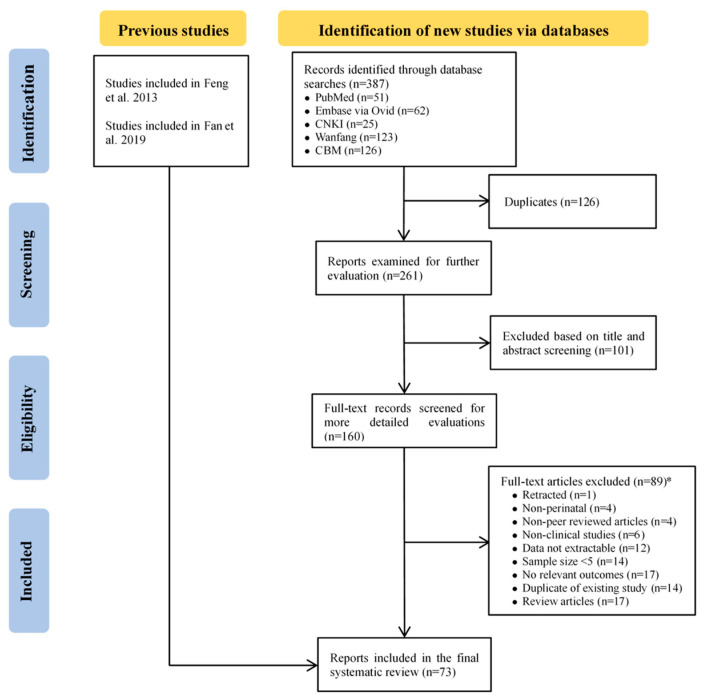
PRISMA flow diagram. * Supplementary Materials S2 presents the studies excluded during the full-text screening and details the reasons for each exclusion.

Figure 1 presents the literature search results and the study selection process. The initial electronic database search yielded 387 records. After removing duplicates, the remaining records underwent title and abstract screening, and 227 records were excluded based on the predefined eligibility criteria. The 160 full-text articles were retrieved and assessed in detail for eligibility, and 89 articles were excluded after full-text review (exclusion reasons listed in Supplementary Materials S2). Ultimately, 71 studies met the inclusion criteria. In addition, we identified two relevant previous systematic reviews that covered the literature up to 2010 and 2017, respectively. We incorporated them into our synthesis, resulting in a total of 73 eligible studies published between 1964 and 2026. These 73 studies collectively provided sufficient data for analysis, encompassing 956 pregnant women and 749 neonates.

### 3.2. Characteristics of the Included Studies

Table 1 summarizes the characteristics of the 73 studies. In terms of study design, the publications comprised two systematic reviews, 58 case series, three case–control studies, and ten cohort studies. Supplementary Materials S3 (Tables S1–S4) presents the detailed quality assessment results for each study type. Overall, the methodological quality of the included studies was moderate, with the mean scores (expressed as a proportion of the maximum possible score) as follows: 8.1 out of 10 for case series, 8.3 out of 10 for case–control studies, 8.5 out of 11 for systematic reviews, and 7.8 out of 11 for cohort studies.

Patient sample sizes varied considerably across studies, ranging from 5 to 204. All data were collected retrospectively (1964–2024), and the majority of the included studies were conducted in hospital settings. Regarding the study populations, 59 studies focused on pregnant women, 61 studies on neonates, and 47 studies included a mixed population of both mothers and neonates.

### 3.3. Epidemiology of Pregnancy-Associated Listeriosis

**Figure 2:**
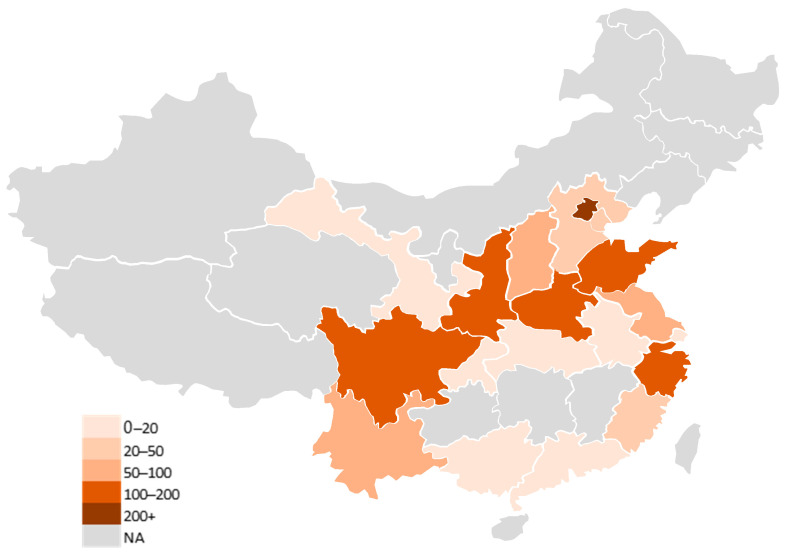
Geographical distribution and number of included studies on pregnancy-related listeriosis.

Pregnancy-associated listeriosis cases were reported in 19 of the 31 mainland Chinese provinces, indicating substantial geographic variation in documented disease occurrence. The highest case counts were observed in Beijing (*n* = 215), Zhejiang (*n* = 143), and Shaanxi (*n* = 142), while no cases were identified in the remaining 12 provinces (Figure 2, Table 1).

**Figure 3:**
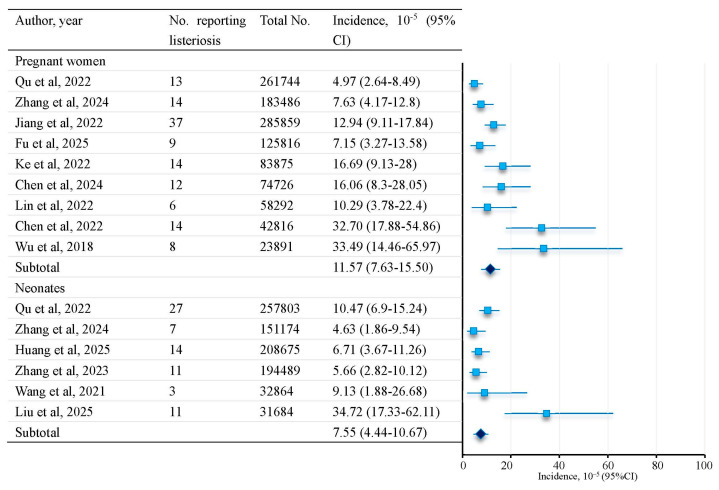
Incidence of listeriosis reported by studies stratified by pregnant women and neonates.

Nine studies reported incidence data for invasive listeriosis among pregnant women; the pooled incidence was 11.57 (95% CI, 7.63–15.50) per 100,000 pregnancies, with substantial between-study heterogeneity (*I*^2^ = 73%). Six studies reported incidence data for neonatal listeriosis; the pooled incidence was 7.55 (95% CI, 4.44–10.67) per 100,000 live births, also with substantial heterogeneity (*I*^2^ = 60%) (Figure 3). The observed heterogeneity across both pooled estimates may be attributed to differences in geographic regions and variations in study periods spanning six decades.

Infections occurred throughout the year, yet they were most frequently reported from spring to autumn, with a peak in summer. Among cases with available seasonal data (*n* = 171), the seasonal distribution was as follows: spring (March–May), 25.7% (*n* = 44); summer (June–August), 39.2% (*n* = 67); autumn (September–November), 26.3% (*n* = 45); and winter (December–February), 8.8% (*n* = 15) (Table 1).

### 3.4. Clinical Characteristics and Outcomes of Pregnancy-Related Listeriosis

#### 3.4.1. Maternal-Fetal Listeriosis

Clinical features and outcomes in pregnant women are detailed in Supplementary Materials S4. The mean age of affected pregnant women was 29.23 ± 4.25 years. Maternal infection occurred across all gestational stages: first trimester (≤13 weeks), 18 cases (3.7%); second trimester (14–27 weeks), 143 cases (29.4%); and third trimester (≥28 weeks), 326 cases (66.9%). A history of consumption of potentially contaminated food was reported in 93 cases (9.7%). Multiple gestation was documented in 20 pregnancies.

Fever was the most frequently reported presenting symptom (513/956, 53.7%), followed by abdominal pain (205/956, 21.4%) and flu-like symptoms (120/956, 12.6%). Laboratory findings most commonly revealed positive cultures from the following sources: blood cultures (415/956, 43.4%), placental cultures (110/956, 11.5%), and amniotic fluid cultures (60/956, 6.3%). Only two maternal deaths were reported during the study period [9,28].

Regarding antibiotic treatment, penicillin-based regimens (alone or in combination with other agents) were administered in 57 cases (6.0%). In contrast, cephalosporins—which are known to be ineffective against *L. monocytogenes*—were used in 311 cases (32.5%). Adverse maternal-fetal outcomes included stillbirth or miscarriage (240/956, 25.1%), premature delivery (208/956, 21.8%), and low birth weight (<2500 g) (109/956, 11.4%). Cesarean section was performed in 101 cases (10.5%).

#### 3.4.2. Neonatal Listeriosis

**Figure 4:**
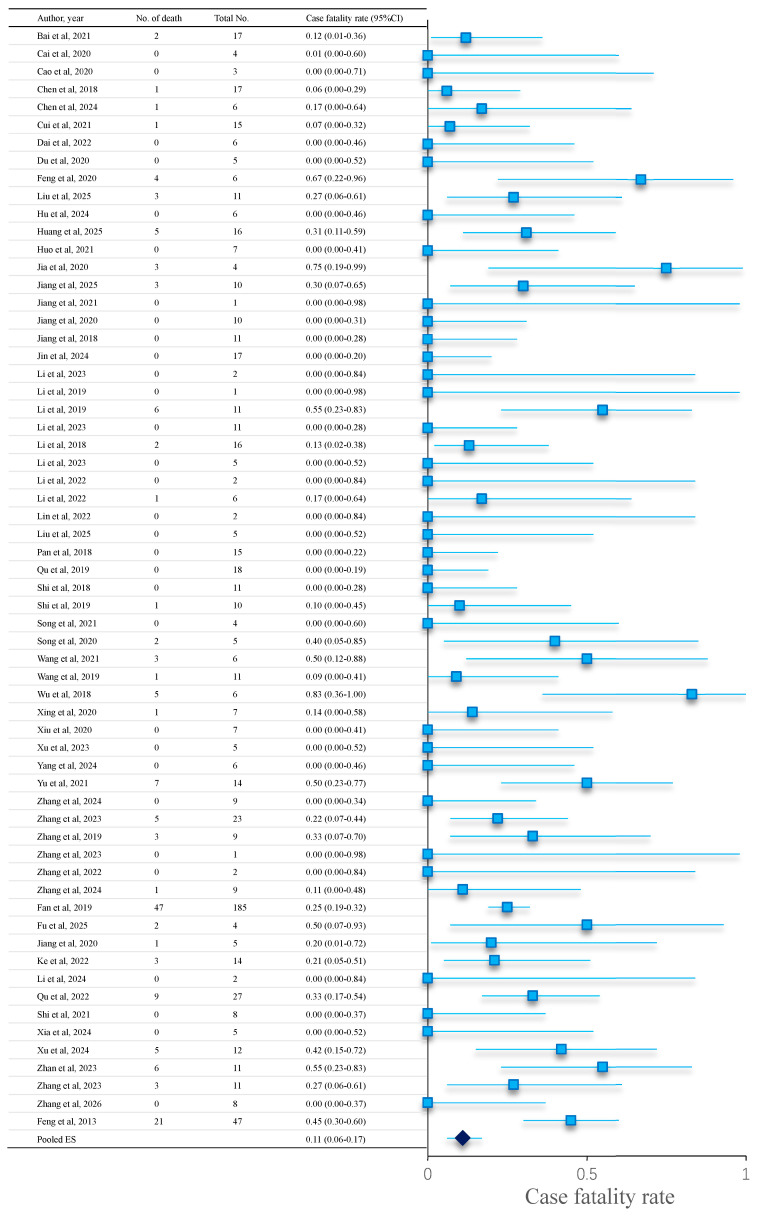
Case fatality rate of neonatal listeriosis.

Clinical characteristics and outcomes of neonatal listeriosis are summarized in Supplementary Materials S5. Among neonatal infections, 94.0% (457/486) presented as early-onset disease, whereas 6.0% (29/486) presented as late-onset disease. The proportion of affected neonates who were female was 46.2% (186/403, with 83 cases missing sex data). The most common symptoms were respiratory distress (261/749, 34.8%) and fever (197/749, 26.3%). Blood culture was positive in 563 specimens (75.1%), and cerebrospinal fluid culture (CSF) was positive in 155 specimens (20.7%). Clinical involvement included sepsis (210/749, 28.0%), cerebral hemorrhage (93/749, 12.4%), and pneumonia (90/749, 12.0%). Penicillin-based antibiotics were administered in 206 cases (27.5%), whereas cephalosporins were used in 117 cases (15.6%). The pooled case fatality rate among neonates with listeriosis was 11% (95% CI, 0.06–0.17; *I*^2^ = 60%) (Figure 4).

### 3.5. Subtypes of Pregnancy-Associated Listeriosis

Serotype data were available for 137 clinical *Listeria* isolates. The most commonly identified serotypes were 1/2b (64/137, 46.7%), followed by 1/2a (50/137, 36.5%) and 4b (23/137, 16.8%). Multilocus sequence typing (MLST) data were available for 113 isolates, revealing 16 distinct sequence types (STs), among which ST87 was the most prevalent (23/113, 20.4%). The full distribution of serotypes and STs is provided in Supplementary Materials S6.

## 4. Discussion

This systematic review, based on 73 studies spanning more than six decades, provides a comprehensive overview of pregnancy-related listeriosis in China and represents one of the largest sample sizes reported to date. Our findings confirm that pregnancy-related listeriosis remains a significant public health concern in China, with substantial geographic variation, a distinct summer seasonal peak, and considerable morbidity burden for both mothers and neonates.

The pooled incidences of obstetric and neonatal listeriosis are higher than those documented in a recent nationwide Spanish study (5.7 per 100,000 pregnancies and 4.7 per 100,000 live births, respectively) [5], but comparable to estimates from the United States (12.0 per 100,000 pregnancies and 8.6 per 100,000 live births, respectively) [86]. This discrepancy may reflect differences in dietary habits, diagnostic capacity, and the use of sentinel hospital surveillance [9]. For instance, the higher Chinese estimates could partly be attributed to the sentinel hospital-based surveillance system, which is designed to capture cases from selected high-volume hospitals and may overrepresent cases relative to true population-based incidence. Conversely, the true population incidence in China is likely higher than routine data indicate, as our included studies predominantly capture hospital data from selected regions and may miss mild or asymptomatic infections that do not present to sentinel facilities. Given the sporadic, widespread, and occasionally asymptomatic nature of listeriosis, a national notification system is warranted [5,7,10,87].

The highest counts in Beijing, Zhejiang, and Shaanxi may reflect either regional differences in population density and healthcare resources or true geographic variations in dietary and environmental exposures. Conversely, the absence of cases in 12 provinces likely indicates surveillance gaps and underdiagnosis rather than disease absence.

The seasonal distribution in our review showed a summer peak (39.2% of cases), consistent with the well-established seasonality of listeriosis [83,88]. This pattern has been attributed to higher ambient temperatures favoring *Listeria* growth in food products, as well as increased consumption of refrigerated and ready-to-eat foods during warmer months. The consistency of this seasonal trend across different geographical regions reinforces the importance of strengthening food safety messaging during warmer seasons, particularly for pregnant women, who should be advised to avoid high-risk foods such as unpasteurized dairy products, deli meats, and pre-prepared salads during summer months.

The clinical presentation of maternal listeriosis in this study was dominated by fever, similar to earlier reports [4,5,7], and did not differ substantially from symptoms caused by other bacterial pathogens. This nonspecific presentation is a key diagnostic challenge: fever in a pregnant woman may be attributed to a wide range of causes, delaying the consideration of listeriosis and the initiation of appropriate empirical therapy. Importantly, maternal mortality was extremely low (only two deaths), mirroring the favorable maternal prognosis observed in both the Spanish study and the MONALISA cohort (zero deaths) [5,7]. This contrasts with the high case fatality rates reported in non-pregnant immunocompromised populations [7,10], underscoring the unique host–pathogen interaction during pregnancy. During pregnancy, the maternal immune system undergoes a shift toward a Th2-dominant response to accommodate the semi-allogeneic fetus, which may paradoxically limit excessive inflammatory damage to the placenta while allowing bacterial proliferation [3,87].

Despite the generally mild maternal disease course, fetal and neonatal outcomes were severe. In the present review, 58.3% of infected pregnant women experienced major adverse pregnancy outcomes (stillbirth, miscarriage, prematurity, or low birth weight), which is consistent with the MONALISA study [7] but substantially higher than the 18.9% reported in the Spanish study [5]. This discrepancy arises because our review and the MONALISA study [7] used infected pregnancies as the denominator, thereby capturing early miscarriages and stillbirths that were excluded from the Spanish analysis [5], which used deliveries as the denominator.

Maternal bacteremia was independently associated with adverse fetal outcomes in the Spanish study [5], and Mylonakis et al. [89] demonstrated that early initiation of appropriate antibiotic therapy significantly reduced fetal loss. Although our data did not permit multivariable adjustment, the high proportion of positive blood cultures (43.4%) strongly suggests that bacteremia may be a key driver of adverse outcomes in Chinese pregnant women. The relatively high rate of positive placental cultures (11.5%) and amniotic fluid cultures (6.3%) further underscores the importance of obtaining microbiological specimens from these sites when listeriosis is suspected, as they may provide diagnostic confirmation even when maternal blood cultures are negative.

Early-onset neonatal listeriosis results from in utero transmission and typically presents as non-specific sepsis, which is further complicated by prematurity [4,5,6]. The neonatal case fatality rate in our review was 11%, which is comparable to the 9.8% reported in the Spanish study [5]. Both rates are considerably lower than those documented in the 2000s [89], reflecting major advances in neonatal intensive care, including improved respiratory support, surfactant therapy, and the widespread use of empirical antibiotic regimens that cover *Listeria* in suspected early-onset sepsis protocols. Nevertheless, a mortality rate of approximately 10% remains unacceptably high. Prematurity is both a consequence of *Listeria* infection and an independent risk factor for neonatal death, creating a vicious cycle in which infection precipitates preterm delivery, and prematurity in turn increases susceptibility to severe complications. Furthermore, the frequent occurrence of respiratory distress (261 of 749 neonates) underscores the need for prompt respiratory support and proactive management of premature infants, although this aspect lies beyond the scope of our review.

With regard to serogroup distribution, serogroup 4b is the predominant cause of human listeriosis in Western countries (e.g., the MONALISA study [7] and Mylène et al. [90]). In East Asia, however, serogroups 1/2b (ST87) and 1/2a account for the majority of cases [9,91], as also observed in our study. These geographical differences, which may stem from distinct dietary habits, could have implications for virulence, clinical presentation, and vaccine development, and thus warrant continued surveillance. Notably, ST87, the most prevalent sequence type in our review, has been increasingly recognized as a dominant lineage in Chinese clinical isolates, warranting further investigation through whole-genome sequencing to characterize its virulence determinants and antimicrobial resistance genes.

From a therapeutic perspective, only 6.0% of mothers and 27.5% of neonates received a penicillin-based antibiotic regimen, reflecting the persistent difficulty of clinically diagnosing pregnancy-associated listeriosis. Notably, cephalosporins—which are ineffective against *Listeria* due to the organism’s lack of penicillin-binding proteins susceptible to this class—were administered in 32.5% of maternal cases and 15.6% of neonatal cases. Similar findings from other studies indicate that inappropriate empirical therapy remains a problem, and delays in recognition and treatment further contribute to poor maternal and neonatal outcomes [5,92]. Current guidelines based on expert opinion recommend ampicillin or amoxicillin, often in combination with gentamicin, for suspected listeriosis in pregnant women and neonates [87]. Educational efforts should therefore be directed at obstetricians and pediatricians to avoid the use of cephalosporins when listeriosis is suspected.

Several limitations should be considered when interpreting this systematic review. First, our analysis is based on retrospective, predominantly single-center studies with high heterogeneity, which may introduce bias toward overrepresentation of severe cases and limit generalizability. The high *I*^2^ values observed in the incidence and case fatality estimates indicate considerable between-study variation, which should be interpreted with caution. Although clinical presentation and serotype-specific virulence deserve more attention, the incompletely and heterogeneously reported data across the primary studies only permitted descriptive analyses, precluding statistical comparisons, multivariable meta-regression, and subgroup analyses. Additionally, the potential overlap between prenatal data (e.g., prematurity) and obstetric outcomes may have led to an overestimation of sample sizes. Third, data extracted from two previous systematic reviews are secondary sources, which may overlap with the current search, potentially leading to an overestimation of the sample sizes. We attempted to mitigate this by cross-verifying original sources, but the possibility of duplication cannot be entirely excluded. Fourth, despite our best efforts, some relevant studies may have been inadvertently omitted, particularly those published in journals not indexed in major databases. In addition, the exclusion of case series with fewer than 5 cases may have further contributed to an underestimation of the true incidence of the disease. Finally, information on antibiotic resistance and long-term follow-up was lacking in the included studies, precluding assessment of emerging resistance patterns and neurodevelopmental outcomes in surviving neonates.

## 5. Conclusions

In conclusion, pregnancy-related listeriosis in China carries a substantial burden and is associated with severe fetal and neonatal outcomes. The persistently low rates of appropriate empirical therapy highlight an urgent need to improve clinical awareness and diagnostic stewardship. Enhanced surveillance, targeted food safety interventions, and antenatal education are urgently needed. Future research should prioritize prospective multicenter studies to establish population-based incidence estimates, genomic analyses of clinical and food isolates to trace transmission routes, and long-term follow-up to assess neurodevelopmental outcomes, thereby informing evidence-based guidelines and reducing the avoidable harm caused by *Listeria*.

## Figures and Tables

**Table 1:** Study characteristics.

Author, Year	Region	Study Type	Study Period	Pregnant Women (*n*)	Neonates (*n*)	Incidence	Seasonality *	JBI Score
Bai et al., [11] 2021	Shanxi	case series	2017–2019	3	17	NA	NA	8/10
Cai et al., [12] 2020	Sichuan	case series	2016–2018	4	4	NA	NA	8/10
Cao et al., [13] 2020	Guangdong	case series	2017–2019	7	3	NA	NA	8/10
Chen et al., [14] 2018	Jiangsu	case series	2008–2018	NA	17	NA	3; 11; 2; 4	8/10
Chen et al., [15] 2024	Zhejiang	case series	2019–2023	8	6	NA	NA	8/10
Chen et al., [16] 2022	Zhejiang	case series	2015–2021	14	NA	14/42,816 (P)	NA	9/10
Chui et al., [17] 2021	Henan	case series	2015–2020	38	NA	NA	NA	8/10
Cui et al., [18] 2021	Shandong	case series	2013–2019	12	15	NA	NA	8/10
Dai et al., [19] 2022	Zhejiang	case series	2016–2020	6	6	NA	0; 3; 2; 1	8/10
Du et al., [20] 2020	Tianjin	case series	2014–2018	NA	5	NA	NA	8/10
Feng et al., [21] 2020	Beijing	case series	2013–2019	9	6	NA	NA	8/10
Hao et al., [22] 2022	Beijing	case series	2016–2018	19	NA	NA	NA	8/10
Liu et al., [23] 2025	Hebei	case series	2021–2022	NA	11	11/31,684 (N)	NA	9/10
Hu et al., [24] 2024	Shanxi	case series	2012–2023	18	6	NA	NA	8/10
Huang et al., [25] 2025	Jiangsu	case series	2012–2023	16	16	14/208,675 (N)	3; 7; 7; 0	9/10
Huo et al., [26] 2021	Beijing	case series	2015–2019	14	7	NA	NA	8/10
Jia et al., [27] 2020	Beijing	case series	2012–2017	6	4	NA	NA	8/10
Jiang et al., [28] 2025	Yunnan	case series	2015–2024	30	10	NA	NA	8/10
Jiang et al., [29] 2021	Beijing	case series	2016–2018	7	1	NA	NA	8/10
Jiang et al., [30] 2022	Beijing	case series	2017–2019	37	NA	37/285,859 (P)	NA	9/10
Jiang et al., [31] 2020	Shandong	case series	2014–2019	4	10	NA	NA	8/10
Jiang et al., [32] 2018	Zhejiang	case series	2013–2017	5	11	NA	NA	8/10
Jin et al., [33] 2021	Zhejiang	case series	2015–2020	14	NA	NA	NA	8/10
Jin et al., [34] 2024	Sichuan	case series	2017–2021	20	17	NA	NA	8/10
Li et al., [35] 2023	Beijing	case series	2012–2022	8	2	NA	NA	8/10
Li et al., [36] 2019	Fujian	case series	2015–2017	5	1	NA	NA	8/10
Li et al., [37] 2019	Sichuan	case series	2011–2018	14	11	NA	NA	8/10
Li et al., [38] 2023	Shanxi	case series	2016–2020	13	11	NA	0; 3; 10; 0	8/10
Li et al., [39] 2018	Henan	case series	2014–2017	NA	16	NA	NA	8/10
Li et al., [40] 2023	Henan	case series	2016–2020	7	5	NA	NA	8/10
Li et al., [41] 2022	Shandong	case series	2016–2020	5	2	NA	NA	8/10
Li et al., [42] 2022	Beijing	case series	2017–2019	6	6	NA	NA	8/10
Lin et al., [43] 2022	Guangdong	case series	2014–2020	6	2	6/58,292 (P)	1; 2; 2; 1	9/10
Liu et al., [44] 2025	Shandong	case series	2019–2023	8	5	NA	4; 1; 3; 0	8/10
Pan et al., [45] 2018	Yunnan	cohort study	2014–2016	NA	15	NA	1; 4; 7; 2	8/11
Qu et al., [46] 2019	Shaanxi	case series	2011–2018	18	18	NA	NA	8/10
Shi et al., [47] 2018	Shanxi	case series	2013–2016	NA	11	NA	NA	8/10
Shi et al., [48] 2019	Beijing, Hebei, Henan, Gansu, Fujian	case series	2011–2018	16	10	NA	NA	8/10
Song et al., [49] 2021	Shandong	case series	2018–2019	7	4	NA	NA	8/10
Song et al., [50] 2020	Hebei	case series	2016–2019	5	5	NA	NA	7/10
Sun et al., [51] 2024	Beijing	cohort study	2017–2020	6	NA	NA	NA	6/11
Wang et al., [52] 2021	Guangxi	case series	2015–2017	NA	6	3/32,864 (N)	NA	9/10
Wang et al., [53] 2019	Henan	case series	2012–2017	NA	11	NA	NA	8/10
Wu et al., [54] 2018	Shaanxi	case series	2013–2017	8	6	8/23,891 (P)	NA	9/10
Xing et al., [55] 2020	Henan	case series	2014–2017	11	7	NA	NA	8/10
Xiu et al., [56] 2020	Fujian	case series	2010–2019	8	7	NA	NA	7/10
Xu et al., [57] 2023	Beijing	case series	2017–2022	10	5	NA	NA	8/10
Yang et al., [58] 2024	Shaanxi	case series	2012–2022	8	NA	NA	NA	8/10
Yang et al., [59] 2024	Shanxi	case series	2020–2023	10	6	NA	NA	8/10
Yu et al., [60] 2021	Jiangsu	case series	2011–2020	NA	14	NA	9; 4; 1; 0	8/10
Zhang et al., [61] 2024	Beijing, Ningxia, Jiangsu, Guangdong	case series	2011–2023	6	9	NA	NA	8/10
Zhang et al., [62] 2023	Tianjin	cohort study	2016–2023	NA	23	NA	NA	10/11
Zhang et al., [63] 2019	Shaanxi	case series	2010–2018	NA	9	NA	NA	8/10
Zhang et al., [64] 2023	Beijing	cohort study	2010–2021	12	NA	NA	NA	10/11
Zhang et al., [65] 2022	Shandong	case series	2016–2021	12	2	NA	NA	8/10
Zhang et al., [66] 2024	Shandong	cohort study	2012–2021	14	9	14/183,486 (P), 7/151,174 (N)	NA	8/11
Zheng et al., [67] 2020	Zhejiang	case series	2014–2020	6	NA	NA	NA	8/10
Chen et al., [68] 2024	Shanghai	case–control	2017–2021	12	NA	12/74,726 (P)	NA	8/10
Fan et al., [83] 2019	NA	systematic review	2011–2017	204	185	NA	NA	8/11
Fu et al., [69] 2025	Fujian	case series	2015–2023	9	4	9/125,816 (P)	4; 4; 0; 1	9/10
Jiang et al., [70] 2020	NA	cohort study	2015–2018	NA	5	NA	NA	9/11
Ke et al., [71] 2022	Zhejiang	case–control	2013–2021	14	14	14/83,875 (P)	NA	8/10
Li et al., [72] 2024	Beijing	case series	2016–2021	7	2	NA	NA	8/10
Qu et al., [73] 2022	Shaanxi	cohort study	2011–2020	13	27	13/261,744 (P), 27/257,803 (N)	14; 16; 7; 3	6/11
Shi et al., [74] 2021	Anhui	cohort study	2003–2021	2	8	NA	1; 3; 2; 2	7/11
Xia et al., [75] 2024	Chongqing	cohort study	2012–2022	NA	5	NA	NA	9/11
Xu et al., [76] 2022	Zhejiang	case series	2013–2020	16	NA	NA	4; 9; 2; 1	8/10
Xu et al., [77] 2024	Shaanxi	case–control	2011–2023	23	12	NA	NA	9/10
Zhan et al., [78] 2023	Sichuan	case series	2016–2022	28	11	NA	NA	8/10
Zhang et al., [79] 2023	Hubei	cohort study	2015–2022	NA	11	11/194,489 (N)	NA	6/11
Zhang et al., [80] 2021	Beijing	case series	2014–2018	63	NA	NA	NA	8/10
Zhang et al., [81] 2026	Zhejiang	case series	2016–2022	15	8	NA	NA	8/10
Feng et al., [9] 2013	NA	systematic review	1964–2010	30	47	NA	NA	9/11

Note: * Values are presented in the order Spring, Summer, Autumn, and Winter, separated by semicolons. Abbreviations: NA, not available; P, pregnant; N, neonates.

## Data Availability

All data generated or analyzed during this study are included in this published article.

## Supplementary Materials

The following supporting information can be downloaded at: https://www.mdpi.com/article/10.3390/jcm15155915/s1. Supplementary Materials S1. Search strategy. Supplementary Materials S2. Studies excluded during full text screening. Supplementary Materials S3. JBI Critical Appraisal Checklist. Supplementary Materials S4 and S5. Characteristics of Included Studies. Supplementary Materials S6. Distribution of serotypes and multilocus sequence typing. Supplementary Materials S7. PRISMA 2020 Checklist.
