# Supplementary material for: Listeriosis in Pregnant Women and Neonates in China: A Systematic Review
Source: J Clin Med. 2026 Jul 29;15(15):5915. doi: 10.3390/jcm15155915 (PMC13466708; doi:10.3390/jcm15155915)
Supplement: Supplementary file 1 [file jcm-15-05915-s001.zip › Supplementary Materials S1.pdf]

S1. Search strategy

Table S1. Search strategy in PubMed

Item	Query	Results
#1	"Listeria monocytogenes"[Mesh] OR "Listeria monocytogenes"[tw] OR "L. monocytogenes"[tw]	25,864
#2	"Listeriosis"[Mesh] OR "Listeriosis"[tw] OR "Listeria infection"[tw] OR "Infection due to Listeria monocytogenes"[tw]	10,634
#3	"Pregnancy"[Mesh] OR "Pregnancy Complications"[Mesh] OR pregnan*[tw] OR maternal[tw] OR gestation*[tw] OR prenatal[tw] OR antenatal[tw] OR perinatal[tw] OR postpartum[tw]	1,560,583
#4	"Infant, Newborn"[Mesh] OR "Infant, Newborn, Diseases"[Mesh] OR neonat*[tw] OR newborn*[tw] OR infant*[tw] OR "new born"[tw]	1,810,394
#5	"China"[MeSH Terms] OR "China"[All Fields] OR "Chinese"[All Fields] OR "Anhui"[All Fields] OR "Fujian"[All Fields] OR "Gansu"[All Fields] OR "Guangdong"[All Fields] OR "Guizhou"[All Fields] OR "Hainan"[All Fields] OR "Hebei"[All Fields] OR "Heilongjiang"[All Fields] OR "Henan"[All Fields] OR "Hubei"[All Fields] OR "Hunan"[All Fields] OR "Jiangsu"[All Fields] OR "Jiangxi"[All Fields] OR "Jilin"[All Fields] OR "Liaoning"[All Fields] OR "Qinghai"[All Fields] OR "Shaanxi"[All Fields] OR	4,081,652

	"Shandong"[All Fields] OR "Shanxi"[All Fields] OR "Sichuan"[All Fields] OR "Yunnan"[All Fields] OR "Zhejiang"[All Fields] OR "Beijing"[All Fields] OR "Chongqing"[All Fields] OR "Shanghai"[All Fields] OR "Tianjin"[All Fields] OR "Guangxi"[All Fields] OR "Inner Mongolia"[All Fields] OR "Ningxia"[All Fields] OR "Tibet"[All Fields] OR "Xinjiang"[All Fields].	
#6	("Animals"[Mesh] NOT "Humans"[Mesh])	5,434,896
#7	(#1 OR #2) AND (#3 OR #4) AND #5 NOT #6.<br>Filters: from 2018/1/1 - 2026/3/14	51

Table S2. Search strategy in Embase via Ovid

Item	Query	Results
#1	(Listeria monocytogenes or l monocytogenes or listeriosis or listeria infection).mp.	36130
#2	(pregnan* or matern* or gestation* or antenatal or prenatal or perinatal or peri natal or peri-natal or postnatal or post-natal or postpartum or post partum or post-partum or natal or gravidit* or gravida* or multigravid* or primigravid* or parturition or nullip* or multip* or (newborn* or infan*)).mp.	6228524
#3	(China or Chinese).mp.	1044522
#4	(animal cell or animal experiment or animal model or animal tissue or nonhuman).mp.	9046148
#5	(#1 AND #2 AND #3) NOT #4<br>Limit to yr= 2018/1/1 - 2026/3/14	62

Table S3. Search strategy for Chinese databases

Database	Query	Results
CBM	("M="[All Fields] AND "Listeria"[All Fields] OR "Listeria"[All Fields] OR "Listeria"[All Fields] OR "Listeria"[All Fields] OR "Listeria"[All Fields] OR ("listeria"[All Fields] OR "Listeria"[All Fields] OR "Listeria"[MeSH Terms])) AND ("M="[All Fields] AND ("Perinatal Period"[All Fields] OR "Peripartum Period"[All Fields] OR "Peripartum Period"[All Fields] OR "Peripartum"[All Fields] OR "Perinatal Women"[All Fields] OR "Perinatal Period"[MeSH Terms]) OR ("Mothers"[All Fields] OR "Mothers"[All Fields] OR "Mothers' Club"[All Fields] OR "Mothers"[MeSH Terms]) OR "Mother"[All Fields] OR ("Pregnant Women"[All Fields] OR "Pregnant Women"[All Fields] OR "Pregnant Women"[All Fields] OR "Pregnant Women"[MeSH Terms]) OR "Parturient Women"[All Fields] OR ("Pregnancy"[All Fields] OR "Pregnancy"[All Fields] OR "Pregnancy"[All Fields] OR "Pregnancy"[MeSH Terms]) OR ("Pregnancy"[All Fields] OR "Pregnancy"[All Fields] OR "Pregnancy"[All Fields] OR "Pregnancy"[MeSH Terms]) OR ("Parturition"[All Fields] OR "Parturition"[All Fields] OR "Fetal Delivery"[All Fields] OR "Birth"[All Fields] OR "Pregnancy and Childbirth"[All Fields] OR "Parturition"[MeSH Terms]) OR ("Postpartum"[All Fields] OR "Postpartum Period"[All Fields] OR "Postpartum Period"[All Fields] OR "Postpartum Women"[All Fields] OR "Puerperium"[All Fields] OR "Postpartum Period"[MeSH Terms]) OR "Antepartum"[All Fields])	126
CNKI	(AB%= Listeria OR Listeria OR Listeria OR Listeria OR Listeria OR listeria) AND (AB%= Perinatal Period OR Mothers OR Mother OR Pregnant Women OR Parturient Women OR Pregnancy OR Pregnancy OR Parturition OR Postpartum OR Antepartum)	25
Wanfang	Abstract: (Listeria OR Listeria OR Listeria OR Listeria OR listeria OR Listeria) AND (Perinatal Period OR Mothers OR Mother OR Pregnant Women OR Parturient Women OR Pregnancy OR Pregnancy OR Parturition OR Postpartum OR Antepartum)	123
