# Supplementary material for: Listeriosis in Pregnant Women and Neonates in China: A Systematic Review
Source: J Clin Med. 2026 Jul 29;15(15):5915. doi: 10.3390/jcm15155915 (PMC13466708; doi:10.3390/jcm15155915)
Supplement: Supplementary file 1 [file jcm-15-05915-s001.zip › Supplementary Materials S2.pdf]

S2. Studies excluded during full text screening

Studies	Reason for exclusion
Chen L, et al. 2021	Review articles
Chen S, et al. 2020	Duplicate of existing study
Chen W, et al. 2019	Sample size <5
Cheng L, et al. 2019	Data not extractable
Duan R, et al. 2019	Duplicate of existing study
Fan Z, et al. 2018	Review articles
Fan Z, et al. 2019	Sample size <5
Guan H, et al. 2021	Review articles
Guo J, et al. 2023	Data not extractable
Guo L, et al. 2024	Non-clinical studies
Hao G, et al. 2023	Review articles
Hu G, et al. 2025	Sample size <5
Hu J, et al. 2025	Sample size <5
Jing X, et al. 2019	Duplicate of existing study
Kuang L, et al. 2022	Duplicate of existing study
Li C, et al. 2018	Non-perinatal
Li C, et al. 2019	Review articles
Li C, et al. 2022	Non-clinical studies
Li H, et al. 2020	Data not extractable
Li K, et al. 2024	Review articles
Li M, et al. 2019	Sample size <5
Li W, et al. 2018	Duplicate of existing study
Li W, et al. 2019	Duplicate of existing study
Li W, et al. 2020	Review articles
Li W, et al. 2022	Review articles
Li W, et al. 2023	Duplicate of existing study
Li X, et al. 2020	Sample size <5
Li X, et al. 2025	Non-peer reviewed articles
Li Y, et al. 2018	Review articles
Lin H, et al. 2024	No relevant outcomes

Studies	Reason for exclusion
Lin N, et al. 2018	No relevant outcomes
Lin W, et al. 2025	Sample size <5
Liu C, et al. 2025	Non-clinical studies
Liu L, et al. 2025	Data not extractable
Liu X, et al. 2025	No relevant outcomes
Liu Y, et al. 2025	Non-perinatal
Liu Y, et al. 2025	Data not extractable
Liu Z, et al. 2025	Duplicate of existing study
Lu L, et al. 2020	Data not extractable
Lu T, et al. 2024	Non-peer reviewed articles
Lu X, et al. 2021	Non-perinatal
Luo Q, et al. 2022	Non-peer reviewed articles
Luo Y, et al. 2019	Duplicate of existing study
Luo Y, et al. 2021	Data not extractable
Luque-Sastre L, et al. 2018	Review articles
Lyu S, et al. 2023	Review articles
Niu Y, et al. 2022	Data not extractable
Niu Y, et al. 2024	Data not extractable
Qiu H, et al. 2019	No relevant outcomes
Rahardiyan D, et al. 2019	No relevant outcomes
Shang L, et al. 2025	Review articles
Shi T, et al. 2025	Non-clinical studies
Ssentongo E, et al. 2025	No relevant outcomes
Sun W, et al. 2019	No relevant outcomes
Sun Y, et al. 2023	Review articles
Sun Z, et al. 2022	No relevant outcomes
Wang C, et al. 2021	No relevant outcomes
Wang T, et al. 2022	No relevant outcomes
Wang T, et al. 2025	Duplicate of existing study
Wang Y, et al. 2022	Sample size <5

Studies	Reason for exclusion
Wang Y, et al. 2024	Data not extractable
Wang Z, et al. 2021	Duplicate of existing study
Wei C, et al. 2021	Sample size <5
Wei M, et al. 2023	No relevant outcomes
Wei Q, et al. 2018	Sample size <5
Wei Q, et al. 2023	Sample size <5
Wu M, et al. 2022	Review articles
Wu T, et al. 2020	Duplicate of existing study
Xiao L, et al. 2018	Review articles
Xing F, et al. 2022	No relevant outcomes
Xu W, et al. 2024	Non-perinatal
Yan H, et al. 2023	Review articles
Yang L, et al. 2024	Non-peer reviewed articles
Yang S, et al. 2020	Sample size <5
Yang X, et al. 2021	Retracted
Yao L, et al. 2019	Data not extractable
Zhang E, et al. 2026	Non-clinical studies
Zhang J, et al. 2021	No relevant outcomes
Zhang J, et al. 2022	No relevant outcomes
Zhang J, et al. 2023	Sample size <5
Zhang J, et al. 2024	Sample size <5
Zhang X, et al. 2019	Duplicate of existing study
Zhang Y, et al. 2024	No relevant outcomes
Zhang Z, et al. 2025	Duplicate of existing study
Zhao C, et al. 2022	Review articles
Zhao Q, et al. 2024	Non-clinical studies
Zhao X, et al. 2018	No relevant outcomes
Zhou J, et al. 2022	No relevant outcomes
Zhu J, et al. 2022	Data not extractable

References

1. Chen L, et al. Progress in research on tumor immunotherapy using attenuated Listeria monocytogenes as a vector. Chinese Journal of Agricultural Biotechnology 2021, 29(8):1622-1629.

2. Chen S, et al. Epidemiology of Human Listeriosis in China during 2008-2017. Foodborne Pathogens and Disease 2020, 17(2):119-125.

3. Chen W, et al. Molecular type analysis of Listeria monocytogenes strains isolated from clinical cases and food source in Fujian, China, 2016. Chinese Journal of Zoonoses 2019, 35(2):145-148.

4. Cheng L, et al. Pathogens and clinical features of preterm infants with sepsis. Chinese Journal of Contemporary Pediatrics 2019, 21(9):881-885.

5. Duan R, et al. Perinatal outcome in women with bacterial sepsis: A cross-sectional study from West China. Medicine 2019, 98(44):e17751.

6. Fan Z, et al. Advances in traceability analysis methods for Listeria and their application. Chinese Journal of Internal Medicine 2018, 57(11).

7. Fan Z, et al. Clinical and etiological analysis of listeriosis cases. Chinese Journal of Infection and Chemotherapy 2019, 19(5):524-529.

8. Guan H, et al. Research progress on biological toxicological characteristics and control methods of Listeria monocytogenes. Agricultural Products Processing (First Half) 2021(4):71-76,79.

9. Guo J, et al. Evaluation of microbiological epidemiology and clinical characteristics of maternal bloodstream infection: a 10 years retrospective study. Frontiers in Microbiology 2023, 14:1332611.

10. Guo L, et al. Clostridiales in the Gut Against Listeria monocytogenes Infection Through Growth Inhibition. Foodborne Pathogens and Disease 2024, 21(4):248-256.

11. Hao G, et al. Research progress on virulence genes and pathogenic mechanisms of Listeria monocytogenes. Chinese Journal of Food Hygiene 2023, 35(3):481-486.

12. Hu G, et al. Maternal-Fetal Listeriosis in China: Clinical and Genomic Characteristics From an ST8 Listeria monocytogenes Case. Infection and Drug Resistance 2025, 18:1313-1324.

13. Hu J, et al. Clinical characteristics, pathogens and prognosis of neonatal sepsis with different onset times. Journal of Guizhou Medical University 2025, 50(4):580-586.

14. Jing X, et al. Listeriosis in mainland China: A systematic review. Open Forum Infectious Diseases 2019, 6(Supplement 2):S588.

15. Kuang L, et al. Analysis of listeriosis infection cases during pregnancy among 70,131 deliveries. Journal of Obstetrics and Gynaecology Research 2022, 48(1):66-72.

16. Li C, et al. Clinical characteristics and etiology of bacterial meningitis in Chinese children >28 days of age, January 2014-December 2016: A multicenter retrospective study. International Journal of Infectious Diseases 2018, 74:47-53.

17. Li C, et al. Diagnosis and treatment analysis of pregnancy complicated with Listeria monocytogenes infection. Chinese Medical Journal 2019, 54(4):361-363.

18. Li C, et al. The potential role and regulatory mechanism of IL-33/ST2 axis on T lymphocytes during lipopolysaccharide stimulation or perinatal Listeria infection. International Immunopharmacology 2022, 108:108742.

19. Li H, et al. Early-onset and late-onset neonatal sepsis: clinical characteristics analysis. Chinese Journal of Maternal and Child Health Research 2020, 31(4):502-505.

20. Li K, et al. Research progress on the effect of Listeria infection on pregnancy in humans and ruminants. Chinese Journal of Zoonoses 2024, 40(7):656-661.

21. Li M, et al. Clinical characteristics and next generation sequencing of three cases of Listeria monocytogenes meningitis with complications. Chinese Journal of Pediatrics 2019, 57(8):603-607.

22. Li W, et al. Sentinel listeriosis surveillance in selected hospitals, China, 2013-2017. Emerging Infectious Diseases 2019, 25(12):2274-2277.

23. Li W, et al. The Epidemiology of Listeria monocytogenes in China. Foodborne Pathogens and Disease 2018, 15(8):459-466.

24. Li W, et al. Whole-Genome Sequencing-Based Characterization of Clinical Listeria monocytogenes Isolates in China, 2013-2019. Foodborne Pathogens and Disease 2023, 20(4):158-168.

25. Li W, et al. Research progress on pregnancy failure caused by Listeria monocytogenes infection. Life Sciences 2020, 32(6):614-620.

26. Li W, et al. Research progress in detection technology of Listeria monocytogenes. Chinese Journal of Health Laboratory Technology 2022, 32(22).

27. Li X, et al. Nosocomial cross-infection of hypervirulent Listeria monocytogenes sequence type 87 in China. Annals of Translational Medicine 2020, 8(9):603.

28. Li X, et al. Pregnant women beware! Listeria hides on the dining table and poses great harm. Family Medicine 2025(21):65.

29. Li Y, et al. Listeria monocytogenes and pyroptosis. Chinese Animal Husbandry and Veterinary Digest 2018, 34(1):69-70.

30. Lin H, et al. Proteomic Analysis of Vibrio parahaemolyticus-Stimulated Pinctada martensii Proteins for Antimicrobial Activity, Potential Mechanisms, and Key Components. Antibiotics 2024, 13(11):1100.

31. Lin N, et al. Survey on awareness of listeriosis among pregnant women in Chaoyang District, Beijing. Chinese Journal of Health Education 2018, 34(5):459-461.

32. Lin W, et al. A cross-sectional study on clinical vigilance in the diagnosis and treatment of listeriosis among pregnant women and their knowledge, attitudes, and practices regarding listeriosis in Gansu Province, China. Frontiers in Public Health 2025, 13:1661344.

33. Liu C, et al. Function analysis of LIPI-4 in Listeria monocytogenes reveals a key role of flagella formation in the regulation of virulence. Virulence 2025, 16(1):2543144.

34. Liu L, et al. Epidemiological characteristics and disease burden of bacterial meningitis in hospitalized children in China: a 6-year nationwide retrospective study. BMC Infectious Diseases 2025, 25(1):592.

35. Liu X, et al. Quantitative risk assessment of Listeria monocytogenes in pre-packaged non-vacuum refrigerated ready-to-eat cooked meat products in Chengdu. Journal of Sichuan University (Medical Sciences) 2025, 56(1):239-246.

36. Liu Y, et al. Clinical, molecular, and resistance features of Listeria monocytogenes in non-perinatal patients with listeriosis: 8-year retrospective data from four tertiary hospitals in Shandong, China. PeerJ 2025, 13(3):e19126.

37. Liu Y, et al. Multicenter study on clinical characteristics of neonatal bacterial meningitis. Chinese Journal of Neonatology 2025, 40(7).

38. Liu Z, et al. Clinical characteristics and drug resistance genes of 11 neonates with Listeria monocytogenes septicemia. Chinese Journal of Nosocomiology 2025, 35(14):2149-2153.

39. Lu L, et al. Pathogens responsible for early-onset sepsis in Suzhou, China. Japanese Journal of Infectious Diseases 2020, 73(2):148-152.

40. Lu T, et al. A foodborne disease that cannot be ignored: listeriosis in pregnancy. Food and Life 2024(1):32-34.

41. Lu X, et al. Analysis of 57 cases of invasive infection with Listeria monocytogenes. Chinese Journal of Laboratory Medicine 2021, 44(5):421-425.

42. Luo Q, et al. The ‘killer’ in the refrigerator: Listeria that is not afraid of cold. Family Medicine (Part 2) 2022(8):56.

43. Luo Y, et al. Comparative analysis of maternal and neonatal outcomes of perinatal Listeria infection. Guangdong Medical Journal 2019, 40(S1):306-308.

44. Luo Y, et al. Etiological analysis of early-onset bacterial infection in neonates. International Medicine and Health Guidance News 2021, 27(16).

45. Luque-Sastre L, et al. Antimicrobial Resistance in Listeria Species. Microbiology Spectrum 2018, 6(4).

46. Lyu S, et al. Expert consensus on detection and prevention of foodborne Listeria monocytogenes infection. Chinese Journal of Public Health Engineering 2023, 22(3):430-432.

47. Niu Y, et al. Risk factors associated with food consumption and food-handling habits for sporadic listeriosis: a case-control study in China from 2013 to 2022. Emerging Microbes & Infections 2024, 13(1):2307520.

48. Niu Y, et al. Risk factors for sporadic listeriosis in Beijing, China: A matched case-control study. Epidemiology and Infection 2022, 150:e62.

49. Qiu H, et al. Diagnosis and treatment of acute colonic pseudo-obstruction after cesarean section. Chinese Journal of Coloproctology 2019, 39(10):17-19.

50. Rahardiyan D, et al. Antibacterial potential of catechin of tea (Camellia sinensis) and its applications. Food Research 2019, 3(1):1-6.

51. Shang L, et al. Recent Advances in Host Immune Mechanisms Against Listeria monocytogenes Infection. Journal of Inflammation Research 2025, 18:10821-10833.

52. Shi T, et al. IL-33 Has a Protective Effect Against Perinatal Listeriosis by Regulating Mitophagy of CD8(+) T Lymphocytes. Journal of Interferon and Cytokine Research 2025, 45(8):300-313.

53. Ssentongo E, et al. Antibiotic susceptibility and risk factors for listeriosis in women with spontaneous abortion in Ugandan tertiary hospitals: a cross-sectional study. BMC Pregnancy and Childbirth 2025, 25(1):227.

54. Sun W, et al. Quantitative risk assessment of Listeria monocytogenes in bulk cooked meat from production to consumption in China: a Bayesian approach. Journal of the Science of Food and Agriculture 2019, 99(6):2931-2938.

55. Sun Y, et al. Clinical characteristics analysis of listeriosis during pregnancy. Medical Theory and Practice 2023, 36(16):2736-2738.

56. Sun Z, et al. Aetiological Characteristics of Infectious Diarrhea in Yantai City, Shandong Province, China in 2017. Viruses 2022, 14(2):216.

57. Wang C, et al. Analysis of Pathogen Distribution and Its Antimicrobial Resistance in Bloodstream Infections in Hospitalized Children in East China, 2015-2018. Journal

of Tropical Pediatrics 2021, 67(1):fmaa077.

58. Wang T, et al. Morbidity, Mortality, and Epidemiological Features of Clinical Invasive Listeriosis Isolates from Sentinel Surveillance in Beijing, China, 2013-2022. Foodborne Pathogens and Disease 2025, 22(12):873-882.

59. Wang T, et al. Survey on knowledge, attitude and practice related to listeriosis among pregnant women in Beijing. Chinese Journal of Health Education 2022, 38(3):222-227.

60. Wang Y, et al. Genomic epidemiology of hypervirulent Listeria monocytogenes CC619: Population structure, phylodynamics and virulence. Microbiological Research 2024, 280:127591.

61. Wang Y, et al. Nursing care of a patient with advanced pregnancy complicated by Listeria infection and secondary sepsis. Chinese Journal of Critical Care Nursing 2022, 3(4):338-340.

62. Wang Z, et al. An Update Review on Listeria Infection in Pregnancy. Infection and Drug Resistance 2021, 14:1967-1978.

63. Wei C, et al. Clinical characteristics of patients with listeriosis. Journal of Central South University (Medical Sciences) 2021, 46(3):257-262.

64. Wei M, et al. Risk factors for acute colonic pseudo-obstruction after cesarean section. Chinese Journal of Integrated Traditional and Western Medicine Surgery 2023, 29(03):358-361.

65. Wei Q, et al. Etiology analysis and intervention measures of central nervous system infection in premature infants. Chinese Health Care 2023, 41(18):35-38.

66. Wei Q, et al. Pregnancy-related listeriosis and literature review. Chinese Journal of Obstetrics & Gynecology and Pediatrics (Electronic Edition) 2018, 14(02):218-223.

67. Wu M, et al. Placenta-specific virulence factors involved in Listeria monocytogenes infection. Chinese Journal of Biotechnology 2022, 38(6):2139-2152.

68. Wu T, et al. Clinical analysis of 102 cases of pregnancy complicated with Listeria infection in China. Electronic Journal of Clinical Medical Literature 2020, 7(52):9-11.

69. Xiao L, et al. Listeria monocytogenes infection during pregnancy and its impact on mothers and infants. China Medical Herald 2018, 15(15):30-33.

70. Xing F, et al. Listeriosis in a Metropolitan Hospital: Is Targeted Therapy a Risk Factor for Infection? Frontiers in Medicine 2022, 9:888038.

71. Xu W, et al. Clinical characteristics analysis of 25 cases of non-perinatal adult Listeria monocytogenes infection. Chinese Journal of Infectious Diseases 2024, 42(2):84-90.

72. Yan H, et al. Research progress on in vivo and in vitro models of placental infection by Listeria monocytogenes. Chinese Journal of Biotechnology 2023, 39(10):3985-4003.

73. Yang L, et al. Beware of Listeria harming your baby's health. Family Medicine 2024(20):67.

74. Yang S, et al. Value of umbilical cord blood culture in preterm infants with early-onset sepsis. Chinese General Practice 2020, 23(27):3422-3426.

75. Yang X, et al. 56 cases of pregnancy complicated with Listeria monocytogenes infection. Journal of Clinical and Pathological Research 2021, 41(6).

76. Yao L, et al. Risk assessment of intrauterine infection on neonatal asphyxia. China Foreign Medical Treatment 2019, 38(17):30-32.

77. Zhang E, et al. Baicalin inhibits Listeria monocytogenes-induced embryonic loss through the JNK/NEK7-NLRP3/GSDMD pathway. International Immunopharmacology 2026, 168(Pt 2):115921.

78. Zhang J, et al. Analysis of gastrointestinal pathogen infection status in NICU infants within 3 days after birth. Chinese Science and Technology Journal Database (Abstract Edition) Medicine and Health 2023(11).

79. Zhang J, et al. Clinical and Genomic Characteristics of a Clinical Listeria Monocytogenes ST120 Isolate Recovered from a Pregnant Woman. Infection and Drug Resistance 2024, 17:229-237.

80. Zhang J, et al. Rapid and precise identification of bloodstream infections using a pre-treatment protocol combined with high-throughput multiplex genetic detection system. BMC Infectious Diseases 2022, 22(1):823.

81. Zhang J, et al. Semi-quantitative risk assessment of Listeria monocytogenes contamination in bulk cooked meat products sold in Zhejiang Province. Preventive Medicine 2021, 33(7):656-659,664.

82. Zhang X, et al. Isolation and characterization of clinical Listeria monocytogenes in Beijing, China, 2014-2016. Frontiers in Microbiology 2019, 10(MAY):981.

83. Zhang Y, et al. Comparative proteomics study of human chorionic trophoblast cells infected with Listeria monocytogenes. Chinese Journal of Zoonoses 2024, 40(7):628-635.

84. Zhang Z, et al. Epidemiological characteristics of listeriosis cases reported in Chinese literature from 2010 to 2023. Chinese Journal of Zoonoses 2025, 41(06):653-659.

85. Zhao C, et al. Pearls & Oy-sters: Diagnosis and Subtyping of Listeria Ventriculitis in an Immunocompetent Host. Neurology 2022, 99(3):123-126.

86. Zhao Q, et al. Formation and recovery of Listeria monocytogenes in viable but nonculturable state under different temperatures combined with low nutrition and high NaCl concentration. Food Research International 2024, 192:114774.

87. Zhao X, et al. Dietary hygiene behaviors and consumption of high-risk foods for Listeria monocytogenes among pregnant women in Fangshan District, Beijing. Capital Journal of Public Health 2018, 12(2):81-84.

88. Zhou J, et al. Application of metabolomics analysis to aid in understanding the pathogenicity of different lineages and different serotypes of Listeria monocytogenes. International Journal of Food Microbiology 2022, 373:109694.

89. Zhu J, et al. Clinical characteristics analysis of early-onset and late-onset neonatal sepsis. Journal of Developmental Medicine (Electronic Edition) 2022, 10(2):126-131.
