# Supplementary material for: Listeriosis in Pregnant Women and Neonates in China: A Systematic Review
Source: J Clin Med. 2026 Jul 29;15(15):5915. doi: 10.3390/jcm15155915 (PMC13466708; doi:10.3390/jcm15155915)
Supplement: Supplementary file 1 [file jcm-15-05915-s001.zip › Supplementary Materials S3.pdf]

S3. JBI Critical Appraisal Checklist

Table S1: Quality assessment of systematic reviews (n=2) following a JBI critical appraisal checklist

Author, year	Q1	Q2	Q3	Q4	Q5	Q6	Q7	Q8	Q9	Q10	Q11	Overall
Feng et al., 2013	1	1	1	1	0	1	1	1	0	1	1	9/11
Fan et al., 2019	1	1	1	1	0	0	1	1	0	1	1	8/11

Q1. Is the review question clearly and explicitly stated?

Q2. Were the inclusion criteria appropriate for the review question?

Q3. Was the search strategy appropriate?

Q4. Were the sources and resources used to search for studies adequate?

Q5. Were the criteria for appraising studies appropriate?

Q6. Was critical appraisal conducted by two or more reviewers independently?

Q7. Were there methods to minimize errors in data extraction?

Q8. Were the methods used to combine studies appropriate?

Q9. Was the likelihood of publication bias assessed?

Q10. Were recommendations for policy and/or practice supported by the reported data?

Q11. Were the specific directives for new research appropriate?

Table S2: Quality assessment of case series (n=58) following a JBI critical appraisal checklist

Author, year	Q1	Q2	Q3	Q4	Q5	Q6	Q7	Q8	Q9	Q10	Overall
Bai et al, 2021	1	1	1	1	1	0	1	1	1	0	8/10
Cai et al, 2020	1	1	1	1	1	0	1	1	1	0	8/10
Cao et al, 2020	1	1	1	1	1	0	1	1	1	0	8/10
Chen et al, 2018	1	1	1	1	1	0	1	1	1	0	8/10
Chen et al, 2024	1	1	1	1	1	0	1	1	1	0	8/10
Chen et al, 2022	1	1	1	1	1	1	1	1	1	0	9/10
Chui et al, 2021	1	1	1	1	1	0	1	1	1	0	8/10
Cui et al, 2021	1	1	1	1	1	0	1	1	1	0	8/10
Dai et al, 2022	1	1	1	1	1	0	1	1	1	0	8/10
Du et al, 2020	1	1	1	1	1	0	1	1	1	0	8/10
Feng et al, 2020	1	1	1	1	1	0	1	1	1	0	8/10
Hao et al, 2022	1	1	1	1	1	0	1	1	1	0	8/10
Liu et al, 2025	1	1	1	1	1	1	1	1	1	0	9/10
Hu et al, 2024	1	1	1	1	1	0	1	1	1	0	8/10
Huang et al, 2025	1	1	1	1	1	1	1	1	1	0	9/10
Huo et al, 2021	1	1	1	1	1	0	1	1	1	0	8/10
Jia et al, 2020	1	1	1	1	1	0	1	1	1	0	8/10
Jiang et al, 2025	1	1	1	1	1	0	1	1	1	0	8/10
Jiang et al, 2021	1	1	1	1	1	0	1	1	1	0	8/10
Jiang et al, 2022	1	1	1	1	1	1	1	1	1	0	9/10
Jiang et al, 2020	1	1	1	1	1	0	1	1	1	0	8/10
Jiang et al, 2018	1	1	1	1	1	0	1	1	1	0	8/10
Jin et al, 2021	1	1	1	1	1	0	1	1	1	0	8/10
Jin et al, 2024	1	1	1	1	1	0	1	1	1	0	8/10

Li et al, 2023	1	1	1	1	1	0	1	1	1	0	8/10
Li et al, 2019	1	1	1	1	1	0	1	1	1	0	8/10
Li et al, 2019	1	1	1	1	1	0	1	1	1	0	8/10
Li et al, 2023	1	1	1	1	1	0	1	1	1	0	8/10
Li et al, 2018	1	1	1	1	1	0	1	1	1	0	8/10
Li et al, 2023	1	1	1	1	1	0	1	1	1	0	8/10
Li et al, 2022	1	1	1	1	1	0	1	1	1	0	8/10
Li et al, 2022	1	1	1	1	1	0	1	1	1	0	8/10
Lin et al, 2022	1	1	1	1	1	1	1	1	1	0	9/10
Liu et al, 2025	1	1	1	1	1	0	1	1	1	0	8/10
Qu et al, 2019	1	1	1	1	1	0	1	1	1	0	8/10
Shi et al, 2018	1	1	1	1	1	0	1	1	1	0	8/10
Shi et al, 2019	1	1	1	1	1	0	1	1	1	0	8/10
Song et al, 2021	1	1	1	1	1	0	1	1	1	0	8/10
Song et al, 2020	1	1	0	1	1	0	1	1	1	0	7/10
Wang et al, 2021	1	1	1	1	1	1	1	1	1	0	9/10
Wang et al, 2019	1	1	1	1	1	0	1	1	1	0	8/10
Wu et al, 2018	1	1	1	1	1	1	1	1	1	0	9/10
Xing et al, 2020	1	1	1	1	1	0	1	1	1	0	8/10
Xiu et al, 2020	1	1	0	1	1	0	1	1	1	0	7/10
Xu et al, 2023	1	1	1	1	1	0	1	1	1	0	8/10
Yang et al, 2024	1	1	1	1	1	0	1	1	1	0	8/10
Yang et al, 2024	1	1	1	1	1	0	1	1	1	0	8/10
Yu et al, 2021	1	1	1	1	1	0	1	1	1	0	8/10
Zhang et al, 2024	1	1	1	1	1	0	1	1	1	0	8/10
Zhang et al, 2019	1	1	1	1	1	0	1	1	1	0	8/10
Zhang et al, 2022	1	1	1	1	1	0	1	1	1	0	8/10
Zheng et al, 2020	1	1	1	1	1	0	1	1	1	0	8/10
Fu et al, 2025	1	1	1	1	1	1	1	1	1	0	9/10
Li et al, 2024	1	1	1	1	1	0	1	1	1	0	8/10
Xu et al, 2022	1	1	1	1	1	0	1	1	1	0	8/10
Zhan et al, 2023	1	1	1	1	1	0	1	1	1	0	8/10
Zhang et al, 2021	1	1	1	1	1	0	1	1	1	0	8/10
Zhang et al, 2026	1	1	1	1	1	0	1	1	1	0	8/10

Q1. Were there clear criteria for inclusion in the case series?

Q2. Was the condition measured in a standard, reliable way for all participants included in the case series?

Q3. Were valid methods used for identification of the condition for all participants included in the case series?

Q4. Did the case series have consecutive inclusion of participants?

Q5. Did the case series have complete inclusion of participants?

Q6. Was there clear reporting of the demographics of the participants in the study?

Q7. Was there clear reporting of clinical information of the participants?

Q8. Were the outcomes or follow up results of cases clearly reported?

Q9. Was there clear reporting of the presenting site(s)/clinic(s) demographic information?

Q10. Was statistical analysis appropriate?

Table S3: Quality assessment of case-control studies (n=3) following a JBI critical appraisal checklist

Author, year	Q1	Q2	Q3	Q4	Q5	Q6	Q7	Q8	Q9	Q10	Overall
Chen et al, 2024	1	1	1	1	1	0	0	1	1	1	8/10
Xu et al, 2024	1	1	1	1	1	1	0	1	1	1	9/10
Ke et al, 2022	1	0	1	1	1	1	0	1	1	1	8/10

Q1. Were the groups comparable other than the presence of disease in cases or the absence of disease in controls?

Q2. Were cases and controls matched appropriately?

Q3. Were the same criteria used for identification of cases and controls?

Q4. Was exposure measured in a standard, valid and reliable way?

Q5. Was exposure measured in the same way for cases and controls?

Q6. Were confounding factors identified?

Q7. Were strategies to deal with confounding factors stated?

Q8. Were outcomes assessed in a standard, valid and reliable way for cases and controls?

Q9. Was the exposure period of interest long enough to be meaningful?

Q10. Was appropriate statistical analysis used?

Table S4: Quality assessment of cohort studies (n=10) following a JBI critical appraisal checklist

Author, year	Q1	Q2	Q3	Q4	Q5	Q6	Q7	Q8	Q9	Q10	Q11	Overall
Pan et al, 2018	1	1	1	0	0	1	1	1	1	0	1	8/11
Sun et al, 2024	1	1	1	0	0	1	1	0	0	0	1	6/11
Zhang et al, 2023	1	1	1	1	1	1	1	1	1	0	1	10/11
Zhang et al, 2023	1	1	1	1	1	1	1	1	1	0	1	10/11
Zhang et al, 2024	1	1	1	0	0	1	1	1	1	0	1	8/11
Jiang et al, 2020	1	1	1	1	0	1	1	1	1	0	1	9/11
Qu et al, 2022	1	1	1	0	0	1	1	0	0	0	1	6/11
Shi et al, 2021	1	1	1	1	0	1	1	0	0	0	1	7/11
Xia et al, 2024	1	1	1	1	0	1	1	1	1	0	1	9/11
Zhang et al, 2023	1	1	1	0	0	1	1	0	0	0	1	6/11

Q1. Were the two groups similar and recruited from the same population?

Q2. Were the exposures measured similarly to assign people to both exposed and unexposed groups?

Q3. Was the exposure measured in a valid and reliable way?

Q4. Were confounding factors identified?

Q5. Were strategies to deal with confounding factors stated?

Q6. Were the groups/participants free of the outcome at the start of the study (or at the moment of exposure)?

Q7. Were the outcomes measured in a valid and reliable way?

Q8. Was the follow up time reported and sufficient to be long enough for outcomes to occur?

Q9. Was follow up complete, and if not, were the reasons to loss to follow up described and explored?

Q10. Were strategies to address incomplete follow up utilized?

Q11. Was appropriate statistical analysis used?
