# Supplementary material for: Listeriosis in Pregnant Women and Neonates in China: A Systematic Review
Source: J Clin Med. 2026 Jul 29;15(15):5915. doi: 10.3390/jcm15155915 (PMC13466708; doi:10.3390/jcm15155915)
Supplement: Supplementary file 1 [file jcm-15-05915-s001.zip › Supplementary Materials S6.pdf]

S6. Distribution of serotypes and multilocus sequence typing

Author, year	Serotype	Multilocus sequence typing (MLST)
Huo et al, 2021	1/2a (n=5), 1/2b (n=7), 4b (n=2), 4d (n=2), 4e (n=2), 3a (n=5), 3b (n=7)	ST87 (n=3), ST1 (n=1), ST121 (n=2), ST8 (n=2), ST5 (n=2), ST59 (n=1), ST155 (n=1), ST2 (n=1), ST403 (n=1)
Wang et al, 2021	1/2a (n=1), 4b (n=4)	ST1 (n=1), ST20 (n=1)
Fan et al, 2019	1/2a (n=6), 1/2b (n=12), 4b (n=3)	ST87 (n=3), ST1 (n=2), ST3 (n=2), ST778 (n=1)
Chen et al, 2024	1/2a (n=10), 1/2b (n=1), 4b (n=1)	NA
Zhang et al, 2021	1/2a (n=21), 1/2b (n=35), 4b (n=7)	ST8 (n=8), ST87 (n=13), ST5 (n=11), ST619 (n=5), ST121 (n=4), others (n=22)
Zhang et al, 2026	1/2a (n=7), 1/2b (n=9), 4b (n=6), 3a (n=1)	ST87 (n=4), ST378 (n=2), ST3 (n=2), ST2 (n=2), ST224 (n=2), ST7 (n=2)

Abbreviation: NA, not available.
